# Novel Therapies, Residual Risk, Familial Hypercholesterolemia, and Digital Tools: Multispecialty Insights from a Dyslipidemia Management Survey

**DOI:** 10.3390/jcm15114205

**Published:** 2026-05-29

**Authors:** António Mesquita-Lousada, Antónia Rocha-Melo-Sousa, Carolina Teixeira, Tiago Rodrigues Guimarães, Mário Marques-Vieira, José Paulo Andrade, Hugo Ribeiro, Manuel Neiva-Sousa, João Rocha-Neves

**Affiliations:** 1 Faculty of Medicine, University of Porto, Alameda Professor Hernâni Monteiro, 4200-319 Porto, Portugal; 2 MEDCIDS—Department of Community Medicine, Information and Health Decision Sciences, Faculty of Medicine, University of Porto, Rua Dr. Plácido da Costa, 4200-450 Porto, Portugal; 3 Unidade de Saúde Familiar—Barão do Corvo, Unidade Local de Saúde do Vila Nova de Gaia/Espinho, EPE, 4400-037 Vila Nova de Gaia, Portugal; 4 Department of Surgery, Unidade Local de Saúde do Barcelos Esposende, 4754-909 Barcelos, Portugal; 5 Department of Angiology and Vascular Surgery, Unidade Local de Saúde de Braga, 4710-243 Braga, Portugal; 6 RISE-Health, Department of Biomedicine, Faculty of Medicine, University of Porto, 4200-319 Porto, Portugal; 7 Unit of Anatomy, Department of Biomedicine, Faculty of Medicine, University of Porto, 4200-319 Porto, Portugal; 8 Faculty of Medicine, University of Coimbra (FMUC), 3004-504 Coimbra, Portugal; 9 Palliative Care Study Center, Faculty of Medicine, University of Coimbra, 3000-548 Coimbra, Portugal; 10 Community Palliative Care Support Team Gaia, Local Health Unit Gaia and Espinho, 4400-129 Vila Nova de Gaia, Portugal; 11 Centre for Innovative Biomedicine and Biotechnology, 3004-504 Coimbra, Portugal; 12 Functional Unit of Maxillofacial Surgery, Unidade Local de Saúde do Alto Ave (ULSAAVE), 4835-044 Guimarães, Portugal; 13 School of Medicine and Biomedical Sciences, Universidade Fernando Pessoa, 4420-096 Porto, Portugal; 14 Department of Vascular Surgery, Unidade Local de Saúde do Alto Ave, EPE, 4835-044 Guimarães, Portugal

**Keywords:** cardiovascular risk assessment, lipoprotein(a), PCSK9 inhibitors, LDL-C, apolipoprotein B, familial hypercholesterolemia, digital decision-support

## Abstract

**Introduction/Objectives**: Although contemporary cardiovascular guidelines endorse intensive low-density lipoprotein cholesterol (LDL-C) lowering and advanced lipid biomarkers for refined risk stratification, important gaps in knowledge and implementation persist. This study evaluated clinician familiarity with novel lipid-lowering therapies, approaches to residual cardiovascular risk, confidence in identifying familial hypercholesterolemia (FH), and perceived usefulness of digital decision-support tools. **Methods**: A multispecialty, cross-sectional online survey (October 2025) of physicians involved in dyslipidemia care was conducted. The questionnaire assessed familiarity, accessibility, and barriers regarding proprotein convertase subtilisin/kexin type 9 (PCSK9) inhibitors, inclisiran, and bempedoic acid; confidence and practice in managing complex populations and residual risk; confidence in detecting FH and expectations for lipoprotein(a) [Lp(a)]; and perceived value of digital decision-support and automated risk alerts. Descriptive statistics and chi-square tests were performed. **Results**: Ninety-five clinicians completed the survey, with the largest group (41.1%) being general practitioners. Of them, 20.0% reported familiarity with all three novel therapies, and 49.5% reported restricted access due to cost and reimbursement constraints. Overall confidence in managing dyslipidemia in complex populations was moderate. Of note, 31.6% did not routinely assess residual risk after achieving LDL-C targets. Among those who did, imaging-based evaluation of subclinical atherosclerosis was the most frequently selected approach, followed by Lp(a) and triglycerides, hs-CRP, and apoB. Confidence in recognizing FH was modest, and expectations regarding future Lp(a) testing differed across specialties. Most respondents endorsed integrated decision-support tools and automated risk-alert prompts. **Conclusions**: Implementation gaps persist in dyslipidemia care, while strong receptiveness to digital decision-support highlights an opportunity to align practice more closely with evidence-based recommendations.

## 1. Introduction

Nearly one-quarter of cardiovascular events are directly attributable to elevated low-density lipoprotein cholesterol (LDL-C) levels [1]. The causal role of LDL-C in the pathogenesis of Atherosclerotic Cardiovascular Disease (ASCVD) has been unequivocally established through converging evidence from epidemiological cohorts, randomized clinical trials, and Mendelian randomized studies [2,3]. The 2025 Focused Update of the European Society of Cardiology/European Atherosclerosis Society (ESC/EAS) guidelines on dyslipidemia management emphasizes increasingly stringent LDL-C targets stratified by cardiovascular risk category and reinforces the role of combining lipid-lowering therapy to achieve these goals [4].

Beyond conventional statins and ezetimibe, the therapeutic armamentarium for dyslipidemia has expanded considerably with the introduction of novel agents, namely, proprotein convertase subtilisin/kexin type 9 (PCSK9) inhibitors (evolocumab and alirocumab) [5], the small-interfering RNA inclisiran, and bempedoic acid [6,7,8,9]. These therapies have demonstrated substantial additional LDL-C reductions in landmark clinical trials, with PCSK9 inhibitors lowering LDL-C by approximately 50–60% [6,7], inclisiran by 40–51% [8], and bempedoic acid by 17–21% [9,10], thereby expanding the possibilities for achieving guideline-recommended targets [11]. However, despite strong evidence supporting their efficacy, real-world adoption of these agents remains limited by cost and reimbursement constraints, administrative complexity in prescription processes, and variable clinician familiarity with their prescription indications and use criteria [12].

In parallel, a critical challenge in contemporary cardiovascular prevention is managing patients who remain at elevated cardiovascular risk despite achieving LDL-C targets [13]. Residual risk may be driven by elevated lipoprotein(a) [Lp(a)], apolipoprotein B (apoB), triglyceride-rich remnant lipoproteins, or subclinical systemic inflammation, all of which contribute independently to atherosclerotic burden [4,14,15,16,17,18,19]. Current ESC/EAS guidelines recommend a once-in-a-lifetime assessment of Lp(a) and apoB, recognizing their independent contributions to cardiovascular risk beyond conventional lipid parameters [4]. Despite this, prior survey data suggest that most clinicians do not routinely assess these biomarkers, and approaches to residual risk stratification vary markedly across specialties [20,21].

Familial hypercholesterolemia (FH) represents another underaddressed dimension of dyslipidemia management [22]. FH is one of the most common monogenic disorders, affecting approximately 1 in 200–300 individuals in the general population [23]. Yet, it remains severely underdiagnosed worldwide, with fewer than 1% of cases identified in many countries [24] and with the prevalence of FH being unknown in 90% of countries worldwide [25]. Early diagnosis of FH is essential since affected individuals carry a substantially elevated lifetime cardiovascular risk that warrants aggressive lipid-lowering strategies from a young age [26]. Clinician confidence in recognizing and managing FH across different specialties has been insufficiently explored, particularly in multidisciplinary settings where patients frequently first present to non-lipid-focused specialists [24,27,28,29].

Additionally, the role of emerging clinical technologies in dyslipidemia management is gaining attention. Lp(a) testing is increasingly recognized as a valuable tool for cardiovascular risk reclassification [30]. Its integration into routine care is expected to expand as specific Lp(a)-lowering therapies, including antisense oligonucleotides and small interfering RNAs, approach clinical availability [31]. Simultaneously, digital decision-support tools that integrate lipid biomarkers, imaging data, and validated risk calculators into a unified clinical interface have been proposed to bridge the gap between guideline recommendations and clinical practice [32]. Electronic health record-embedded clinical decision support systems have shown promise in improving guideline adherence and therapeutic optimization across various medical domains.

This study aimed to characterize contemporary clinical practice patterns in dyslipidemia management, focusing on the adoption of novel lipid-lowering therapies, clinicians’ approaches to patients who remain at high cardiovascular risk despite achieving LDL-C targets, management of special populations, and identification of familial hypercholesterolemia. The possibility of future integration of Lp(a) testing and digital decision-support tools into routine care was also evaluated.

## 2. Methods

### 2.1. Study Design

This was an observational, descriptive, cross-sectional study based on an anonymous online survey distributed to a convenience sample of physicians from multiple specialties, recruited primarily through professional networks in Portugal, with additional international participation, in October 2025.

### 2.2. Study Population and Recruitment

The study targeted physicians in active clinical practice, residents and specialists alike, working in General Practice/Family Medicine, Cardiology, Endocrinology, Internal Medicine, Vascular Surgery, or other specialties involved in dyslipidemia management. Recruitment proceeded through multiple channels, namely institutional email distribution lists, medical professional societies, and physician-oriented online communities and networks, including WhatsApp® (WhatsApp LLC, Menlo Park, CA, USA) and LinkedIn® (LinkedIn Corporation, Sunnyvale, CA, USA). To qualify for inclusion, respondents had to (i) hold a medical degree and maintain active clinical activity within one of the listed specialties and (ii) complete every mandatory item of the questionnaire. Excluded were (i) non-physician healthcare professionals (such as nurses, pharmacists, dietitians, and nutritionists), (ii) physicians whose clinical work did not encompass patients with dyslipidemia, and (iii) duplicate submissions, which were identified through metadata review.

### 2.3. Survey Instrument

A pilot study was carried out involving 10 participants: college members, cardiologists, and primary care physicians with regular experience managing patients with dyslipidemia, to assess the questionnaire’s feasibility and clarity. Feedback collected during this phase informed targeted revisions to improve comprehensibility and relevance.

Most of the items employed Likert-type, single-choice, or multiple-choice response formats, with no logic-based branching between questions. Respondents were expected to complete the survey in approximately 8 min (Supplementary Material—File S1).

### 2.4. Data Collection

Survey responses were collected via Google Forms^®^ (Google LLC, Mountain View, CA, USA) and distributed via Web 2.0 platforms. Before engaging with the questionnaire, all prospective participants were presented with an informed consent statement detailing the study aims, the entirely voluntary nature of participation, and the unconditional right to withdraw at any point. No personally identifiable information was requested or retained. Anonymity was maintained throughout, and all procedures complied with the requirements of the General Data Protection Regulation (GDPR). Response data were stored on a secure, access-restricted server accessible exclusively to the principal investigator. Upon survey closure, all records were transferred to a password-protected institutional repository and permanently deleted from the original platform, in accordance with applicable academic data retention standards (a maximum 5-year retention period). Reporting followed the Checklist for Reporting Results of Internet E-Surveys (CHERRIES) guidelines [33].

### 2.5. Statistical Analysis

Initial data handling and preliminary descriptive summaries were performed in Microsoft 365 Excel version 16.108.3 (Microsoft Corp., Redmond, WA, USA). Since completion of all survey items was mandatory for submission, no missing data were present. Results from the pilot phase were used exclusively to refine the instrument and were not included in the final dataset.

Statistical analyses were carried out using IBM SPSS Statistics version 30.0 (IBM Corp., Armonk, NY, USA; release 2025). Participant characteristics and survey responses were summarised through descriptive statistics. Categorical variables were expressed as absolute frequencies and proportions. The responses were stratified according to medical specialty. Differences between specialties in categorical variables were evaluated using chi-square tests; *p* < 0.05 was considered statistically significant. Fisher’s exact test was used when expected cell counts were less than 5, preserving the validity of inferential comparisons across subgroups. Consistent with the descriptive and exploratory scope of the study, neither formal hypothesis testing nor multivariate modeling was undertaken. Specialty-stratified comparisons are accordingly regarded as hypothesis-generating, given that the modest cell sizes in some subgroups limit the precision and stability of the resulting estimates.

### 2.6. Ethical Considerations

The study was approved by the Ethics Committee of the Faculdade de Medicina da Universidade do Porto (protocol no. 356/CEFMUP-RISEHealth/2025) and conducted in accordance with the Declaration of Helsinki and the GDPR. Prior to accessing the questionnaire, participants confirmed their informed consent through a mandatory checkbox, which summarised the study’s purpose and clarified that withdrawal was permitted at any point. Participation was unpaid and anonymous; no personally identifiable data were collected, and all responses were handled in strict confidence throughout.

## 3. Results

### 3.1. Participant Characteristics

The survey was completed by 95 physicians during October 2025. The majority were practicing in Portugal (*n* = 79, 83.2%), with the remaining respondents distributed across other European countries—Italy, Romania, and Luxembourg (*n* = 9, 9.5%)—Latin America, comprising Brazil, Argentina, and Mexico (*n* = 4, 4.2%), North Africa/Middle East (*n* = 1, 1.1%), South Asia (*n* = 1, 1.1%), and Oceania (*n* = 1, 1.1%).

Given the marked geographic concentration of the sample and the limited representation of other regions, geographic subgroup analyses were not performed. Subsequent inferential analyses, therefore, focused exclusively on comparisons across medical specialties.

General Practice/Family Medicine was the most represented specialty (*n* = 39, 41.1%). Cardiology, Internal Medicine, and Vascular Surgery each contributed 14 respondents (*n* = 14, 14.7% each; combined 44.2%), while Endocrinology accounted for the smallest group (*n* = 9, 9.5%). With respect to weekly clinical exposure to dyslipidemia, the most frequently reported patient volume was 6–10 patients per week (*n* = 38, 40.0%), followed by 11–20 (*n* = 28, 29.5%), 0–5 (*n* = 14, 14.7%), 21–30 (*n* = 8, 8.4%), and 31–40 (*n* = 7, 7.4%) (Table 1).

### 3.2. Familiarity with and Accessibility to Novel Lipid-Lowering Therapies

Overall familiarity with PCSK9 inhibitors, inclisiran, and bempedoic acid was heterogeneous and differed significantly across medical specialties (*p* < 0.001). While 20.0% of respondents reported familiarity with the indications and criteria for all three agents, 38.9% reported awareness without confidence in the prescription criteria, and 16.8% reported no familiarity. Full familiarity tended to be more frequent among cardiologists (50.0%) and endocrinologists (33.3%), whereas general practitioners most commonly reported uncertainty regarding formal indications (46.2% aware but unsure of criteria). Given the small absolute numbers within the endocrinology subgroup, this point estimate should be interpreted with caution.

Perceived accessibility to these therapies was limited in most cases. Nearly half of clinicians (49.5%) reported access to these agents as “restricted”, and 15.8% reported that they were not accessible in their practice. Only 3.2% considered them very accessible. Accessibility differed significantly across specialties (*p* = 0.003), with cardiologists predominantly reporting limited access (92.9%) and internal medicine/geriatrics specialists reporting moderate accessibility more frequently (71.4%).

The most frequently reported barriers to prescribing these agents were cost or lack of reimbursement (47.4%), administrative complexity (28.4%), and limited clinical experience or comfort (27.4%). Significant inter-specialty differences were observed for cost-related barriers and familiarity complexity (both *p* < 0.001). A substantial proportion of cardiologists (92.9%) and endocrinologists (77.8%) reported not routinely prescribing these therapies.

These differences across specialties in familiarity, accessibility, and reported prescribing barriers are detailed in Table 2.

### 3.3. Management of Special Populations and Residual Risk Assessment

Clinicians demonstrated only moderate confidence in managing dyslipidemia among special populations, including patients with autoimmune disease, premature menopause, or polycystic ovary syndrome. Overall, 50.5% of respondents reported being “somewhat confident,” 18.9% “very confident,” 17.9% indicated they would need to review the literature, and 12.6% reported not being confident. Although no statistically significant differences were observed across specialties (*p* = 0.124), endocrinologists most frequently reported higher confidence levels, whereas general practitioners more commonly selected “somewhat confident.”

Regarding clinical adaptation for special conditions, practices were heterogeneous. While 41.1% of clinicians reported adjusting therapy “often” and 9.5% “always,” a substantial proportion (22.1%) “never” or “rarely” modified their approach despite elevated risk profiles inherent to these populations. These patterns again showed no significant specialty-level differences (*p* = 0.173), indicating a systematic, rather than discipline-specific, gap in individualized dyslipidemia management. In patients achieving LDL-C targets but perceived as remaining at high cardiovascular risk, 31.6% of clinicians reported not routinely performing additional assessment. Among those who performed additional residual risk assessment, imaging-based evaluation of subclinical atherosclerosis was the most frequently selected approach (23.1%), followed by biochemical markers including Lp(a) and triglycerides (each 11.5%), hs-CRP (6.3%), and apoB (5.3%), with no significant inter-specialty differences (*p* = 0.248) (Table 3).

### 3.4. Identification of Familial Hypercholesterolemia and Future Role of Lp(a)

Clinician confidence in identifying familial hypercholesterolemia (FH) was moderate but far from optimal, reflecting an important diagnostic gap in contemporary dyslipidemia care. Across the cohort, only 20.0% of respondents felt “very confident”, and an additional 32.6% “confident”, whereas 36.8% reported being only “somewhat confident”. A minority expressed limited confidence (10.6%), with 1.1% reporting no confidence at all. Although cardiologists and endocrinologists tended to report higher self-efficacy, each with one-third of respondents identifying as “very confident”, these differences did not reach statistical significance (*p* = 0.077), suggesting limitations in FH recognition across all specialties (Table 4).

Expectations regarding the future integration of Lp(a) testing demonstrated marked heterogeneity and significant differences across disciplines (*p* < 0.001). Overall, 25.3% of clinicians anticipated that Lp(a) testing would become routine for all adults, while the largest proportion (46.3%) expected testing to remain confined to selected high-risk groups, reflecting growing but still selective awareness of its prognostic relevance as novel Lp(a)-lowering therapies approach clinical availability. A further 26.3% were uncertain about its forthcoming role (Table 4), and 2.1% did not consider it beneficial. Selective high-risk testing was endorsed by 10 of 14 cardiologists (71.4%), whereas universal implementation was projected by 6 of 9 endocrinologists (66.7%); the latter estimate, derived from a small subgroup, warrants cautious interpretation. General practitioners, the largest stratum, displayed the greatest distributional spread, with responses spanning all categories.

### 3.5. Perceived Usefulness of Digital Decision-Support Tools

**Figure 1:**
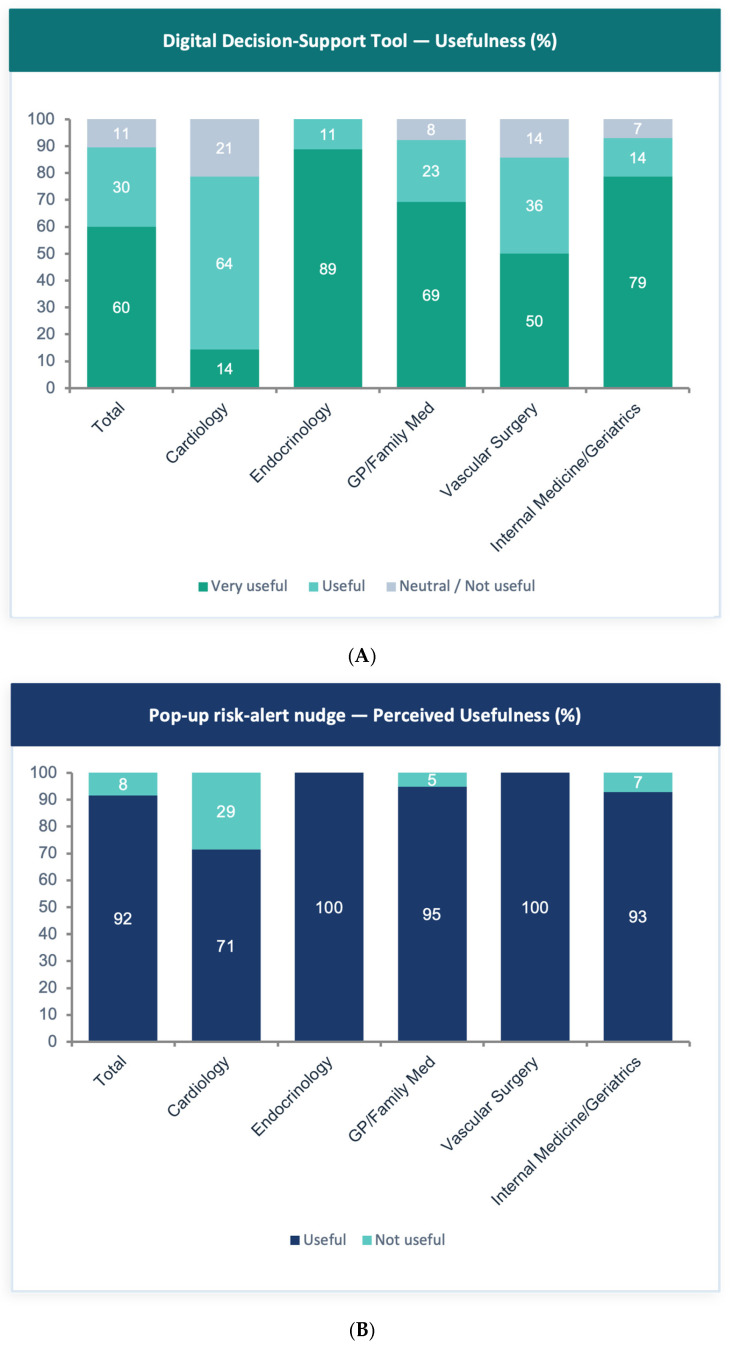
(**A**) Percentage of physicians in each specialty who reported finding a digital decision-support tool integrating lipid markers, imaging, and risk calculators “useful” or “very useful” in clinical practice, stratified by medical specialty. *p* < 0.001. (**B**) Percentage of physicians who considered pop-up risk alerts highlighting patient risk level and lipid targets useful in clinical practice, stratified by medical specialty. *p* = 0.053. Differences between specialties were assessed using the chi-square test for categorical variables. Statistical significance was defined as *p* < 0.05.

A large majority of clinicians perceived value in digital decision-support systems integrating lipid markers, imaging findings, and risk calculators: 60.0% considered such tools “very useful” and 29.5% “useful”, with significant inter-specialty differences (*p* < 0.001). Endocrinologists (88.9%) and internal medicine/geriatrics specialists (78.6%) were the strata in which ‘very useful’ ratings were most frequent, although the small denominators in these subgroups limit the precision of these point estimates (Figure 1A).

Similarly, 91.6% of respondents considered pop-up risk alerts highlighting lipid targets and cardiovascular risk levels to be useful in clinical practice. However, this did not differ significantly across specialties (*p* = 0.053) (Table 5) (Figure 1B).

## 4. Discussion

### 4.1. Main Findings

This multispecialty survey exposes several critical implementation gaps in modern dyslipidemia management. Clinicians reported limited familiarity with novel lipid-lowering therapies and restricted access driven primarily by reimbursement and administrative barriers. Residual cardiovascular risk was inconsistently assessed, with substantial underuse of apoB, Lp(a), and imaging-based tools despite strong guideline endorsement. Confidence in identifying FH remained only moderate across all specialties, highlighting persistent challenges in detecting genetically mediated risk. In contrast, there was widespread enthusiasm for digital decision-support tools and automated risk alerts, indicating that clinicians recognize the need for systems strengthening to improve consistency and guideline adherence. Collectively, the present results delineate a clear implementation gap in contemporary dyslipidemia management, not predominantly due to a lack of awareness, but mainly to uneven familiarity, structural access barriers, and inconsistent integration of residual risk strategies into clinical practice.

### 4.2. Novel Lipid-Lowering Therapies: From Evidence to Access Barriers

Familiarity with PCSK9 inhibitors, inclisiran, and bempedoic acid was limited, as only 20% of respondents were knowledgeable about all three agents, and 16.8% were entirely unfamiliar with them. Given that these therapies represent major advances in dyslipidemia management, PCSK9 inhibitors achieve ~60% LDL-C reduction [6,7], inclisiran offers convenient twice-yearly dosing [8], and bempedoic acid provides a non-statin option [10,12], this knowledge gap is concerning.

More problematically, even among knowledgeable physicians, access was severely restricted: only 3.2% considered these therapies very accessible, and cost or lack of reimbursement was the predominant barrier (47.4%), followed by administrative complexity (28.4%). This finding reflects broader global challenges in access to innovative cardiovascular therapies due to reimbursement [34].

The accessibility issue has profound equity implications. Patients who fail to achieve targets with statins and ezetimibe, including those with familial hypercholesterolemia (prevalence 1:200–300 with <1–10% diagnosed) [23], statin intolerance (overall prevalence of 9.1%) [35], or very high cardiovascular risk (>20% 10-year ASCVD risk), are precisely those who would derive the greatest absolute benefit from PCSK9 inhibitors (~60% additional LDL-C reduction and number needed to treat as low as 20–30 to prevent one major CV event over 5 years) [7]. Restrictive reimbursement policies, while intended to control costs, may paradoxically increase long-term healthcare expenditures by 20–50%, as each prevented myocardial infarction/stroke saves $50,000–$100,000 in acute event costs while averting recurrent events [31,36].

Addressing these barriers requires multi-level interventions: (1) education through society-endorsed guidelines, webinars, and problem-based learning; (2) simplified prescribing pathways reducing administrative burden; (3) value-based pricing negotiations with stakeholders; and (4) advocacy to policymakers emphasizing cost-effectiveness and clinical benefit data from trials such as FOURIER, ODYSSEY OUTCOMES, and CLEAR Outcomes [6,7,9].

While the economic consequences of restricted access to novel lipid-lowering therapies have been explored in other healthcare contexts, our data does not permit country-specific or system-level cost inferences. The reimbursement barriers identified in our survey are consistent with patterns reported in the broader literature, but their economic implications in the Portuguese or international context require dedicated health economic analyses beyond the scope of this study.

### 4.3. Special Populations and Familial Hypercholesterolemia

Management of complex patient groups remains inconsistent. Physicians reported only moderate confidence in treating dyslipidemia among individuals with autoimmune diseases, premature menopause, or polycystic ovary syndrome; conditions associated with inflammation and/or hormonal imbalance that serve to amplify atherosclerotic risk even when LDL-C appears to be controlled [37]. This highlights the persistent underestimation of residual cardiovascular risk in nontraditional contexts and the lack of dedicated programs.

Residual risk assessment was unevenly applied: one-third of clinicians ceased evaluation once LDL-C targets were achieved. Imaging-based evaluation of subclinical atherosclerosis was the most frequently selected residual risk assessment strategy, while Lp(a), though the most commonly used lipid biomarker in this context, and apoB remained underutilised relative to guideline recommendations, revealing a gap between guideline awareness and practical implementation. Despite ApoB being the preferred measurement to refine the estimate of ASCVD risk, in patients with elevated Lp(a), apoB may considerably underestimate risk because Mendelian randomization studies show that the atherogenicity of Lp(a) is approximately 7-fold greater than that of LDL on a per ApoB particle basis [38]. In subjects with increased Lp(a), the association between LDL-cholesterol and incident coronary heart disease (CHD) is increased. Still, the association between apoB and incident CHD is diminished or even lost [39].

Converging data from epidemiological cohorts, Mendelian randomization studies, and post-hoc trial analyses have since demonstrated that apolipoprotein B (apoB), as a direct measure of total atherogenic particle number, outperforms LDL-C in predicting cardiovascular risk, particularly in patients with insulin resistance, metabolic syndrome, or elevated triglycerides, where LDL-C may substantially underestimate atherogenic burden [15,16,17]. Simultaneously, the recognition of genetic contributors, most prominently familial hypercholesterolemia, but also polygenic risk, has reinforced that cardiovascular risk is not solely a product of acquired lifestyle factors but is substantially predetermined at the molecular level. The low rates of apoB utilization (9.5%) and modest confidence in FH recognition documented in our survey suggest that, while awareness of this paradigm shift is growing, its translation into routine clinical practice remains incomplete across all specialties surveyed.

Regarding FH, this condition remains underrecognized, with less than 1% being diagnosed in most countries. All reported studies document failure to achieve recommended LDL cholesterol targets in a large proportion of individuals with FH, and up to 13-fold increased risk of CHD [24]. Although prevalent in roughly 1 in 200 individuals [23], diagnostic confidence was modest, and estimated detection rates remain low worldwide. Delayed identification deprives patients of early lipid-lowering intervention and opportunities for family screening.

Expectations for Lp(a) testing varied markedly across specialties. Approximately one quarter of respondents (25.3%) anticipated universal testing in adults, the largest proportion (46.3%) favoured case-selective testing in high-risk groups, and a further quarter (26.3%) were uncertain, patterns that, while exploratory given the modest specialty-level cell sizes, are consistent with the broader uncertainty surrounding policy and reimbursement structures for emerging biomarkers. Overall, these results demonstrate that awareness of residual risk and FH is widespread but inconsistently applied. Practical integration of apoB and Lp(a) testing, streamlined FH recognition, and support tools embedded in clinical workflows are needed to translate conceptual knowledge into consistent, guideline-based care. In fact, a 2025 intervention at Johns Hopkins Bayview used a 30-min case-based presentation on Lp(a) for internal medicine residents, boosting self-reported confidence in ordering and interpreting Lp(a) from ~30% to >80% and increasing Lp(a) orders >3-fold in the following 3 months [40].

### 4.4. Digital Decision-Support Tools: High Interest, Promising Future

Effective decision-support tools should (1) integrate seamlessly into clinical workflow without adding burden; (2) provide actionable recommendations at the point of care; (3) adapt to individual patient context (comorbidities, prior treatments, contraindications); and (4) offer audit and feedback capabilities [41]. The preference for pop-up alerts suggests that passive reminders may be more acceptable than tools requiring active engagement, though both approaches have demonstrated benefits [42].

The high levels of receptiveness to digital decision-support tools observed in this survey do not reflect actual implementation, usability testing, or demonstrated clinical effectiveness. These findings indicate that clinicians are open to such tools, which justify future implementation and effectiveness studies in real-world clinical workflows.

Implementation challenges include ensuring interoperability with diverse electronic health record systems, maintaining up-to-date guideline algorithms, addressing alert fatigue, and evaluating real-world effectiveness through pragmatic trials [43]. Nonetheless, given the documented gaps in practice revealed by our survey, digital tools represent a scalable, cost-effective intervention strategy [44].

### 4.5. Strengths and Limitations

The present study carries several methodological strengths worthy of acknowledgment. Positioning itself among the first multispecialty surveys conducted predominantly in Portugal, it offers a thorough appraisal of physicians’ knowledge, clinical practices, and perceived barriers across multiple dimensions of dyslipidemia management, encompassing advanced lipid biomarkers such as apolipoprotein B and lipoprotein(a). The inclusion of practitioners from general and family medicine, cardiology, endocrinology, and vascular surgery affords a broad, cross-disciplinary portrait of contemporary clinical approaches to this condition.

That said, several limitations warrant careful consideration. The relatively modest sample (*n* = 95), combined with the cohort’s predominantly Portuguese composition (83.2%), tempers the extent to which these findings can be extrapolated to international clinical contexts. Although the majority of respondents are currently practicing in Portugal, it is worth noting that vascular surgery training in Portugal typically includes structured international residency periods, endowing many such specialists with substantive professional experience in foreign healthcare settings. This circumstance may partially offset concerns regarding external validity and lend broader international resonance to their perspectives, notwithstanding their current practice location. Even so, cross-national heterogeneity in healthcare culture, system organization, and pharmaceutical reimbursement frameworks may shape clinical decision-making beyond the scope of this survey [45,46,47].

In addition, the modest cell sizes within several specialty strata (particularly Endocrinology, *n* = 9) limit the precision of subgroup estimates and the stability of inter-specialty comparisons. Although chi-square and Fisher’s exact tests were applied to preserve inferential validity where expected cell counts were small, the resulting comparisons should be regarded as exploratory and hypothesis-generating; observed differences require confirmation in larger, dedicated multicentre studies before being incorporated into specialty-specific recommendations.

Beyond sample size considerations, recruitment through the authors’ professional networks and social media channels may have systematically underrepresented practitioners outside these spheres or those with limited digital engagement, thereby introducing a potential network selection bias that should be borne in mind when interpreting the results. Additionally, some healthcare professionals who do not engage with Web 2.0 platforms might have been excluded from the survey, further limiting the generalizability of the findings [48]. Third, selection bias is inherent in voluntary online surveys; respondents may be more engaged, knowledgeable, or motivated than non-respondents, potentially overestimating overall awareness and adherence [49]. Fourth, self-report is subject to social desirability bias, with physicians potentially overreporting guideline-concordant behaviors [50].

A pilot phase was conducted with 10 participants to assess the questionnaire’s feasibility and face validity. No formal psychometric validation or reliability assessment was performed. This represents a limitation of the instrument, as the questionnaire was developed specifically for this exploratory study and has not been independently validated.

An important dimension not captured by the present survey is the role of lifestyle modification, dietary counseling, and structured physical activity, which remain the cornerstone of dyslipidemia management and cardiovascular prevention, irrespective of pharmacological therapy. Current ESC/EAS guidelines recommend dietary intervention and regular aerobic exercise as first-line measures for all risk categories, with particular emphasis on patients with diabetic dyslipidemia and metabolic syndrome [4].

Corroborating self-reported data with objective measures, such as prescribing records or lipid goal attainment rates at the patient level, would have strengthened the robustness of the conclusions; however, such validation fell outside the purview of the present study. Additionally, patient-level determinants known to critically shape outcomes of dyslipidemia management, including individual preferences, health literacy, and socioeconomic circumstances, were not captured by the survey instrument [51].

In contrast to the previous survey by Barter et al. [52], which highlighted heterogeneity in dyslipidemia management practices, this study includes a truly multidisciplinary cohort of general practice/family medicine, vascular surgeons, endocrinologists, cardiologists, and internists [52]. These findings support multidisciplinary collaborative care models for dyslipidemia management. Integration of specialty expertise through shared care pathways, teleconsultation for complex cases, and interdisciplinary education can harmonize practices and improve outcomes [53]. Vascular surgery and cardiology patients may particularly benefit from referral to lipid clinics and/or endocrinology for optimization of medical therapy.

## 5. Conclusions

This multispecialty survey reveals substantial implementation gaps in dyslipidemia management, characterized by limited familiarity with novel lipid-lowering agents, restricted access driven by reimbursement and administrative barriers, inconsistent evaluation of residual cardiovascular risk, and only moderate confidence in identifying familial hypercholesterolemia. Despite these shortcomings, clinicians express strong openness to digital decision-support systems that integrate lipid markers, imaging, and risk calculators, suggesting a viable strategy to harmonize care and enhance adherence to evidence-based recommendations.

Addressing the operational barriers highlighted in this study through improved access pathways, targeted professional education, greater use of advanced biomarkers, and digitally enabled clinical workflows will be essential to translating contemporary lipid science into more effective, equitable cardiovascular prevention. Future research should evaluate how systematic incorporation of apoB, Lp(a), and FH screening pathways, supported by automated clinical prompts, influences cardiovascular outcomes and resource allocation across diverse healthcare settings.

## Figures and Tables

**Table 1:** Participant demographics and clinical practice characteristics.

	Total *N* = 95 (%)
**Country/Region**	
Portugal	79 (83.2)
Other European countries	9 (9.5)
Latin America	4 (4.2)
North Africa/Middle East	1 (1.1)
South Asia	1 (1.1)
Oceania	1 (1.1)
**What is your practicing specialty?**	
General Practice/Family	39 (41.1)
Cardiology	14 (14.7)
Internal Medicine/Geriatrics	14 (14.7)
Vascular Surgery	14 (14.7)
Endocrinology	9 (9.5)
Others *	5 (5.3)
**Number of patients with dyslipidemia seen per week**	
0–5	14 (14.7)
6–10	38 (40.0)
11–20	28 (29.5)
21–30	8 (8.4)
31–40	7 (7.4)

Other European countries—Italy, Romania, Luxembourg; Latin America—Brazil, Argentina, Mexico; North Africa/Middle East—Egypt; South Asia—India; Oceania—Australia. Statistical comparisons between specialties were performed using the chi-square test. A *p* < 0.05 was considered statistically significant. * Others include: Nephrology, Oncology, Rheumatology, General Surgery, and Occupational Medicine.

**Table 2:** Familiarity, Accessibility, and Barriers to the Use of Novel Lipid-Lowering Therapies.

	Total *N* (%) *N* = 95	Cardiology *n* = 14	Endocrinology *n* = 9	General Practice/Family Medicine *n* = 39	Vascular Surgery *n* = 14	Internal Medicine/ Geriatrics *n* = 14	*p* Value
**Are you familiar with the indications and criteria for PCSK9 inhibitors, inclisiran, or bempedoic acid?**							
No	16 (16.8)	0 (0.0)	0 (0.0)	8 (20.5)	7 (50.0)	0 (0.0)	<0.001
Aware, but unsure of criteria	37 (38.9)	3 (21.4)	1 (11.1)	18 (46.2)	6 (42.9)	6 (42.9)	
Yes, for some	23 (24.2)	4 (28.7)	5 (55.6)	9 (23.1)	1 (7.1)	3 (21.4)	
Yes, for all three	19 (20.0)	7 (50.0)	3 (33.3)	4 (10.3)	0 (0.0)	5 (35.7)	
**In your practice, how accessible are these therapies for eligible patients?**							
Not accessible	15 (15.8)	0 (0.0)	1 (11.1)	10 (25.6)	3 (21.4)	0 (0.0)	0.003
Limited access	47 (49.5)	13 (92.9)	4 (44.4)	13 (33.3)	5 (35.7)	10 (71.4)	
Only available in secondary prevention	16 (16.8)	1 (7.1)	0 (0.0)	10 (25.6)	5 (35.7)	0 (0.0)	
Moderately accessible	14 (14.7)	0 (0.0)	4 (44.4)	5 (12.8)	1 (7.1)	3 (21.4)	
Very accessible	3 (3.2)	0 (0.0)	0 (0.0)	1 (2.6)	0 (0.0)	1 (7.1)	
**What are the main barriers to prescribing these agents?**							
Cost or lack of reimbursement	45 (47.4)	13 (92.9)	7 (77.8)	13 (33.3)	4 (28.6)	8 (57.1)	<0.001
Administrative complexity	27 (28.4)	6 (42.9)	6 (66.7)	4 (10.3)	3 (21.4)	8 (57.1)	<0.001
Limited experience or comfort with these drugs	26 (27.4)	2 (14.3)	2 (22.2)	12 (30.8)	6 (42.9)	3 (21.4)	0.604
Lack of clear indications	6 (6.3)	1 (7.1)	0 (0.0)	3 (7.7)	1 (7.1)	1 (7.1)	0.953
I do not prescribe them	29 (30.5)	1 (7.1)	0 (0.0)	16 (41.0)	5 (35.7)	3 (21.4)	0.007
Not available	16 (16.8)	0 (0.0)	1 (11.1)	12 (30.8)	3 (21.4)	0 (0.0)	0.028
No perceived added benefit	0 (0.0)	0 (0.0)	0 (0.0)	0 (0.0)	0 (0.0)	0 (0.0)	-

Abbreviations: PCSK9 inhibitors—protein convertase subtilisin/kexin type 9 inhibitors. “Novel therapies” include PCSK9 inhibitors, inclisiran, and bempedoic acid. Differences between specialties were assessed using the chi-square test for categorical variables. Statistical significance was defined as *p* < 0.05.

**Table 3:** Confidence and Clinical Approaches to Special Populations and Residual Risk Stratification.

	Total *N* (%) *N* = 95	Cardiology *n* = 14	Endocrinology *n* = 9	General Practice/Family Medicine *n* = 39	Vascular Surgery *n* = 14	Internal Medicine/ Geriatrics *n* = 14	*p* Value
**How confident are you in managing dyslipidemia in special populations (e.g., patients with autoimmune diseases, post-menopausal women, polycystic ovary syndrome)?**							
I have to review the literature	17 (17.9)	2 (14.3)	1 (11.1)	6 (15.4)	2 (14.3)	4 (28.6)	0.124
Not confident	12 (12.6)	3 (21.4)	0 (0.0)	2 (5.1)	6 (42.9)	1 (7.1)	
Somewhat confident	48 (50.5)	6 (42.9)	5 (55.6)	23 (59.0)	5 (35.7)	7 (50.0)	
Very confident	18 (18.9)	3 (21.4)	3 (33.3)	8 (20.5)	1 (7.1)	2 (14.3)	
**Do you adjust your approach in patients with special conditions (e.g., autoimmune disease, premature menopause)?**							
Never	8 (8.4)	1 (7.1)	0 (0.0)	3 (7.7)	2 (14.2)	1 (7.1)	0.173
Rarely	13 (13.7)	5 (35.7)	1 (11.1)	4 (10.3)	3 (21.4)	0 (0.0)	
Sometimes	26 (27.4)	5 (35.7)	5 (55.6)	10 (25.6)	2 (14.2)	2 (14.3)	
Often	39 (41.1)	3 (21.4)	3 (33.3)	16 (41.0)	7 (7.1)	9 (64.3)	
Always	9 (9.5)	0 (0.0)	0 (0.0)	6 (15.4)	0 (0.0)	2 (14.3)	
**In patients who reach LDL-C targets but still seem high-risk, what do you assess?**							
I do not routinely assess further	30 (31.6)	6 (42.9)	3 (33.3)	14 (35.9)	3 (21.4)	3 (21.4)	0.248
Subclinical atherosclerosis	22 (23.1)	3 (21.4)	0 (0.0)	12 (30.8)	4 (28.6)	2 (14.3)	
Triglycerides	11 (11.5)	2 (14.2)	2 (22.2)	9 (23.1)	3 (21.4)	4 (28.6)	
Inflammatory markers (e.g., hs-CRP)	6 (6.3)	0 (0.0)	0 (0.0)	3 (7.7)	2 (14.2)	0 (0.0)	
Lp(a)	11 (11.5)	2 (14.2)	3 (33.3)	1 (2.6)	2 (14.2)	2 (14.3)	
apoB	5 (5.3)	1 (7.1)	1 (11.1)	0 (0.0)	0 (0.0)	3 (21.4)	

Abbreviations: hs-CRP—high-sensitivity C-reactive protein; Lp(a)—lipoprotein(a); apoB—apolipoprotein B. Differences between specialties were assessed using the chi-square test for categorical variables. Statistical significance was defined as *p* < 0.05.

**Table 4:** Confidence in Identifying Familial Hypercholesterolemia and Future Expectations for Lp(a) Testing.

	Total *N* (%) *N* = 95	Cardiology *n* = 14	Endocrinology *n* = 9	General Practice/Family Medicine *n* = 39	Vascular Surgery *n* = 14	Internal Medicine/Geriatrics *n* = 14	*p* Value
**How confident are you in identifying patients with familial hypercholesterolemia?**							
Not at all confident	1 (1.1)	0 (0.0)	0 (0.0)	0 (0.0)	1 (7.1)	0 (0.0)	0.077
Not confident	9 (9.5)	0 (0.0)	0 (0.0)	1 (2.6)	4 (28.6)	2 (14.3)	
Somewhat confident	35 (36.8)	5 (35.7)	3 (33.3)	20 (51.3)	4 (28.6)	3 (21.4)	
Confident	31 (32.6)	6 (42.9)	3 (33.3)	12 (30.8)	2 (14.3)	6 (42.9)	
Very confident	19 (20.0)	3 (21.4)	3 (33.3)	6 (15.4)	3 (21.4)	3 (21.4)	
**How do you anticipate the role of Lp(a) testing and management will evolve in your clinical practice in the next 5 years?**							
No, I don’t see the benefit	2 (2.1)	2 (14.3)	0 (0.0)	0 (0.0)	0 (0.0)	0 (0.0)	<0.001
Unsure	25 (26.3)	1 (7.1)	1 (11.1)	18 (46.2)	2 (14.2)	1 (7.1)	
Yes, but only in selected high-risk groups	44 (46.3)	10 (71.4)	2 (22.2)	14 (35.9)	8 (57.1)	7 (50)	
Yes, for all adults	24 (25.3)	1 (7.1)	6 (66.7)	7 (17.9)	4 (28.6)	6 (42.9	

Abbreviations: Lp(a)—lipoprotein(a). Differences between specialties were assessed using the chi-square test for categorical variables. Statistical significance was defined as *p* < 0.05.

**Table 5:** Perceived Usefulness of Digital Decision-Support Tools and Risk-Alert Nudges.

	Total *N* (%) *N* = 95	Cardiology *n* = 14	Endocrinology *n* = 9	General Practice/Family Medicine *n* = 39	Vascular Surgery *n* = 14	Internal Medicine/Geriatrics *n* = 14	*p* Value
**Would you find value in a digital decision-support tool that integrates lipid markers, imaging, and risk calculators?**							
Not useful at all	1 (1.1)	0 (0.0)	0 (0.0)	0 (0.0)	0 (0.0)	0 (0.0)	<0.001
Not very useful	2 (2.1)	2 (14.3)	0 (0.0)	0 (0.0)	0 (0.0)	0 (0.0)	
Neutral	7 (7.4)	1 (7.1)	0 (0.0)	3 (7.7)	2 (14.3)	1 (7.1)	
Useful	28 (29.5)	9 (64.3)	1 (11.1)	9 (23.1)	5 (35.7)	2 (14.3)	
Very useful	57 (60.0)	2 (14.3)	8 (88.9)	27 (69.2)	7 (50.0)	11 (78.6)	
**Do you consider pop-up alerts that highlight patient risk level and lipid targets useful in clinical practice?**							
No	8 (8.4)	4 (28.6)	0 (0.0)	2 (5.1)	0 (0.0)	1 (7.1)	0.053
Yes	87 (91.6)	10 (71.4)	9 (100.0)	37 (94.9)	14 (100.0)	13 (92.9)	

Differences between specialties were assessed using the chi-square test for categorical variables. Statistical significance was defined as *p* < 0.05.

## Data Availability

The data supporting the findings of this study are available from the corresponding author upon reasonable request.

## Supplementary Materials

The following supporting information can be downloaded at https://www.mdpi.com/article/10.3390/jcm15114205/s1. File S1: Survey instrument.
