# Supplementary material for: Novel Therapies, Residual Risk, Familial Hypercholesterolemia, and Digital Tools: Multispecialty Insights from a Dyslipidemia Management Survey
Source: J Clin Med. 2026 May 29;15(11):4205. doi: 10.3390/jcm15114205 (PMC13258806; doi:10.3390/jcm15114205)
Supplement: Supplementary file 1 [file jcm-15-04205-s001.zip › jcm-4278121-supplementary.pdf]

Supplemental Materials – File S1: Survey instrument

Novel Therapies, Residual Risk, Familial Hypercholesterolemia, and Digital Tools

Dear colleague,

You are invited to participate in a research study aimed at exploring medical doctors' knowledge, clinical experience, and perceptions regarding the assessment and treatment of dyslipidemia in clinical practice.

This questionnaire, containing 10 questions, is part of a European scientific project that seeks to analyze current practices in lipid profile testing, the therapeutic strategies adopted in response to elevated levels, and the main barriers to its routine implementation in the context of atherosclerotic cardiovascular disease prevention and management.

Participation and Data Handling

Participation in this survey is voluntary and takes approximately 8 minutes. The questionnaire includes multiple-choice and open-ended questions related to your clinical practice and decision-making concerning the use of the lipid profile as a cardiovascular risk marker.

All data will be collected anonymously and used exclusively for scientific purposes. They will be analyzed in aggregate form, with no possibility of individual identification. The study fully complies with the General Data Protection Regulation (GDPR), ensuring confidentiality, security, and data integrity.

All the data will be deleted in August 2026. The investigators declare that they have no conflicts of interest related to this research project.

By proceeding with the questionnaire, you are providing your **informed consent** to participate in this study. You may withdraw at any time or request the deletion of your data by contacting the research team at: **jneves@med.up.pt**

Demographics

Country:

- o Portugal
- o Spain
- o Italy
- o Brazil
- o Other, please specify

What is your medical speciality?

- o Cardiology
- o Endocrinology
- o Internal Medicine
- o General Practice/Family Medicine
- o Nephrology
- o Other, please specify

How many patients do you encounter with dyslipidemia per week in your practice?

- o 0 to 5
- o 6 to 10
- o 11 to 20
- o 21 to 30
- o 31 to 40

Section S1

1. Are you familiar with the indications and criteria for PCSK9 inhibitors, inclisiran, or bempedoic acid?

- No
- Aware, but unsure of criteria
- Yes, for some
- Yes, for all three

2. In your practice, how accessible are these therapies for eligible patients?

- Not accessible
- Limited access
- Only available in secondary prevention
- Moderately accessible
- Very accessible

3. What are the main barriers to prescribing these agents? (You can select multiple answers)

- Cost or lack of reimbursement
- Administrative complexity
- Limited experience or comfort with these drugs
- Lack of clear indications
- I do not prescribe them
- Not available
- No perceived added benefit

Section S2

4. How confident are you in managing dyslipidemia in special populations (e.g., patients with autoimmune diseases, post-menopausal women, polycystic ovary syndrome)?

- I have to review the literature
- Not confident
- Somewhat confident
- Very confident

5. Do you adjust your approach in patients with special conditions (e.g., autoimmune disease, premature menopause)?

- Never
- Rarely
- Sometimes
- Often
- Always

6. In patients who reach LDL-C targets but still seem high-risk, what do you assess? (You can select multiple answers)

- I do not routinely assess further
- Subclinical atherosclerosis
- Triglycerides
- Inflammatory markers (e.g., hs-CRP)
- Lp(a)
- ApoB

Section S3

7. How confident are you in identifying patients with familial hypercholesterolemia?

- Not at all confident
- Not confident
- Somewhat confident
- Confident
- Very confident

8. How do you anticipate the role of Lp(a) testing and management will evolve in your clinical practice in the next 5 years?

- No, I don’t see the benefit
- Unsure
- Yes, but only in selected high-risk groups
- Yes, for all adults

Section S4

9. Would you find value in a digital decision-support tool that integrates lipid markers, imaging, and risk calculators?

- Not useful at all
- Not very useful
- Neutral
- Useful
- Very useful

10. Do you consider pop-up alerts that highlight patient risk level and lipid targets useful in clinical practice?

- Yes
- No

Thank you for your participation.
